# Nonlinear association between visceral adipose tissue area and remnant cholesterol in US adults: a cross-sectional study

**DOI:** 10.1186/s12944-024-02211-z

**Published:** 2024-07-25

**Authors:** Xi Gu, Xun Wang, Sujie Wang, Ying Shen, Leiqun Lu

**Affiliations:** Department of Endocrinology, RuiJin Hospital Lu Wan Branch, Shanghai Jiaotong University School of Medicine, No.149 Chongqing South Road, Shanghai, China

**Keywords:** Remnant cholesterol, Visceral adipose tissue, Nonlinear association, National Health and Nutrition Examination Survey

## Abstract

**Background:**

Excessive visceral adipose tissue (VAT) is associated with a spectrum of diseases, including diabetes, cancer, and cardiovascular diseases. Remnant cholesterol (RC), denoting cholesterol within triglyceride-rich lipoproteins and their metabolic byproducts, has been identified as a key contributor to cardiovascular diseases and related mortality. However, the association between the VAT and RC remains unclear. In this study, the objective is to provide new evidence regarding the association between VAT and RC concentrations.

**Methods:**

4727 individuals aged 18–59 were selected from the National Health and Nutrition Examination Survey conducted between 2011 and 2018 as study participants. This study utilized several weighted linear regression models and a restricted cubic spline (RCS) to explore the association and potential nonlinearities between VAT and RC. Subgroup analyses were performed to determine the consistency of findings.

**Results:**

The mean VAT value was 103.82 ± 1.42 cm^2^, and the median RC value was 18 mg/dl. VAT demonstrated a positive association with RC in a fully adjusted model, with a β and 95% confidence interval (CI) of 0.09 (0.08, 0.11) after adjustment for potential confounders. Analysis using RCS revealed a nonlinear association between the VAT area and RC (*P* < 0.001 for nonlinearity). Adjusted two-piecewise regression models demonstrated β coefficients of 0.13 (95%CI: 0.11 ~ 0.16, *P* < 0.001) for RC in individuals with VAT < 143 cm^2^, and 0.02 (95%CI: -0.01 ~ 0.06, *P* = 0.15) for those with VAT ≥ 143 cm^2^. Interactions were observed among the body mass index (BMI) subgroup; the β coefficients for RC were 0.14 (95%CI: 0.12 ~ 0.16) in those with BMI < 30 kg/m^2^ and 0.05 (95%CI:0.04 ~ 0.07) in those with BMI ≥ 30 kg/m^2^, with a *P*-value of < 0.001 for interaction.

**Conclusions:**

This study identified a nonlinear association between VAT and RC in American adults. Reducing the VAT area may be beneficial in lowering RC concentration, particularly when VAT is < 143 cm^2^ and those with a BMI < 30 kg/m^2^.

**Supplementary Information:**

The online version contains supplementary material available at 10.1186/s12944-024-02211-z.

## Background

Extensive research has established that adipose tissue not only functions as an energy storage site but also as a notable endocrine organ [1]. Excess adipose tissue, particularly visceral adipose tissue (VAT), is implicated in numerous obesity-related disease processes [2]. VAT has been linked to decreased insulin efficiency [3], non-insulin-dependent diabetes [4], as well as their related complications such as diabetic nephropathy and retinopathy [5, 6]. Additionally, excess VAT is associated with a greater probability of developing cancer and a poorer prognosis for colorectal and liver cancers [7, 8]. Importantly, VAT is also associated with a higher prevalence of cardiovascular diseases [9], which are identified as primary contributors to mortality [10].

Very low-density lipoproteins (VLDL), intermediate-density lipoproteins (IDL), and chylomicron remnants jointly form triglyceride-rich lipoproteins (TRLs) [11]. The cholesterol present in TRLs and the products of their metabolism are referred to as remnant cholesterol (RC) [12], which serves as the primary source of lipid-dependent residual risk in cardiovascular diseases [13, 14]. An elevated RC concentration is associated with higher cardiovascular disease mortality [15, 16]. Furthermore, recent studies have revealed additional associations between RC and the presence of non-alcoholic fatty liver disease [17], higher long-term mortality rates in individuals with metabolic dysfunction-associated fatty liver disease [18], new-onset prediabetes [19], and hip bone mineral density [20].

Both VAT and RC play crucial roles as risk factors for the development of cardiovascular diseases; however, their association has not been extensively studied. To address this knowledge gap and examine the hypothesized positive link between the VAT area and RC concentration, a cross-sectional study was carried out, using data from the National Health and Nutrition Examination Survey (NHANES). Unlike previous studies that primarily focused on the individual effects of VAT and RC on cardiovascular health, the direct association between VAT and RC was examined. Additionally, the use of the curve-fitting method facilitated a detailed exploration of potential nonlinear associations, providing new insights into the association between VAT and RC, which remains poorly clarified.

## Methods

### Research subjects

**Fig. 1:**
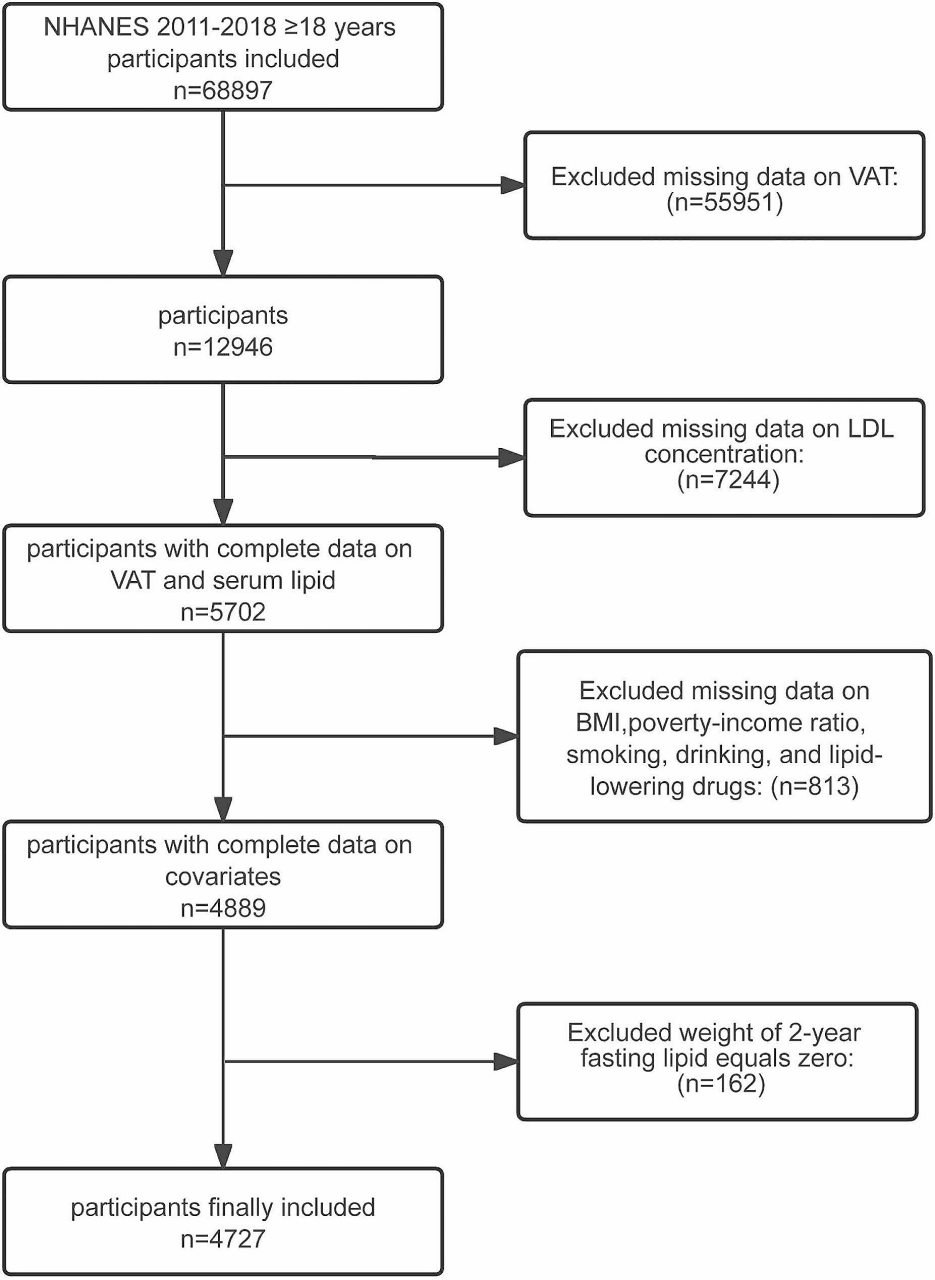
Flowchart of participants’ enrollment from NHANES 2011–2018 Abbreviations: NHANES, National Health and Nutrition Examination Survey; VAT, visceral adipose tissue; LDL, low-density lipoprotein; BMI, body mass index.

During the initial phase, 68,897 participants aged ≥ 18 years were enrolled from the NHANES 2011–2018 dataset. Subsequently, individuals lacking VAT area data and those with missing information on low-density lipoprotein cholesterol (LDL-C) concentrations, poverty-income ratio (PIR), smoking habits, alcohol intake, lipid-lowering drug use, or body mass index (BMI) were excluded. Additionally, individuals with a fasting lipid 2-year weight of zero were excluded from the analysis. Ultimately, the study population comprised 4727 participants (Fig. 1). The NHANES program was conducted by the National Center for Health Statistics (NCHS) and approved by the NCHS Ethics Review Board. The guidelines specified in the Strengthening the Reporting of Observational Studies in Epidemiology statement were strictly followed in this study [21].

### Measurement of RC

Serum samples collected from the NHANES mobile examination center (MEC) were processed, stored, and forwarded to the University of Minnesota, Minneapolis, for analysis. During the MEC visit, the participants were queried regarding their fasting status. Blood samples were collected from individuals who met the 9-h fasting requirement for lipid level assessment. Enzymatic or immunological methods were used to measure the concentrations of total cholesterol (TC), triglycerides (TG), and high-density lipoprotein cholesterol (HDL-C) [22]. The Friedewald formula was applied to calculate LDL-C [23], with TG values of ≤ 400 mg/dl; when TG exceeded this threshold, LDL-C data were considered missing [22]. Finally, the RC was determined by deducting the combined values of LDL-C and HDL-C from the TC [24].

### Measurement of VAT area

The NHANES conducted whole-body dual-energy X-ray absorptiometry (DXA) scans on participants aged 8–59 years who met eligibility requirements. Participants were excluded from the DXA scan if they were pregnant, had used radiographic contrast material (such as barium) within the previous week, weighed more than 450 lb, or were ˃6 feet and 5 inches in height. Scans were performed at the MEC, with VAT defined using the analysis provided by the Hologic APEX software (version 4.0) [25]. The VAT area, which identified the fat within the abdomen, was evaluated between the fourth and fifth lumbar vertebrae. DXA examinations were performed by radiology technologists with proper training and certification [26].

### Covariates

The demographic information used as covariates included age, sex (male or female), and ethnic background (Mexican American, non-Hispanic White, non-Hispanic Black, other Hispanic, and other/multi-racial groups). Additionally, the PIR was divided into low-income (≤ 1.3), middle-income (1.3 to 3.5), and high-income (> 3.5). Educational level was classified as less than high school, high school diploma, and education beyond high school. Lifestyle factors consisted of smoking categories (never, former, current), alcohol consumption (≥ 2 daily drinks for males, ≥ 1 daily drink for females), and physical activity (PA) levels (determined by participation in recreational activities). Body measurements were determined using BMI, which was calculated by dividing weight (kg) by height squared (m^2^). The use of medications to lower lipid levels, including β-hydroxy β-methylglutaryl-CoA reductase inhibitors, cholestyramine, colesevelam, ezetimibe, fenofibrate, gemfibrozil, and niacin, were also considered.

### Statistical analysis

Between December 2023 and May 2024, analyses were performed using NHANES guidelines by considering the intricate sampling design and applying the appropriate sampling weights. Specifically, the sampling weight was equal to one-quarter of the two-year fasting lipid weight. Weighted participant attributes are expressed as mean (standard error) for normally distributed continuous variables and as median (interquartile range [IQR]) for distributions with skewness, whereas categorical data are presented as unweighted numbers and weighted percentages. Differences among VAT tertiles were assessed using χ2 for categorical variables, one-way analysis of variance for normally distributed data, and the Kruskal-Wallis H test for skewed distributions.

Univariate and multivariate linear regression analyses were performed to explore the association between the VAT and RC across the three models. Model 1 was adjusted for age, sex, and ethnicity, while Model 2 was additionally adjusted for educational level, cigarette consumption, alcohol consumption, PIR, and PA. In Model 3, further adjustments were made for BMI and the use of lipid-lowering drugs. Covariates were selected based on available literature [27, 28], clinical judgment, and associations in the univariate analysis (*P* < 0.05). To examine the association of VAT with RC, the VAT area was classified into tertiles and analyzed using multivariate linear regression models.

To account for nonlinear association, a restricted cubic spline (RCS) containing knots positioned at the 5th, 35th, 65th, and 95th distribution points of the exposure distribution in Model 3 was utilized. The likelihood ratio test was employed to evaluate the nonlinearity. If nonlinearity was detected, a two-piecewise linear regression model was constructed around the turning point.

Prespecified analyses of subgroups were performed depending on age (< 40, ≥ 40), sex, ethnicity, and BMI (< 30, ≥ 30) in the adjusted model considering age, sex, ethnicity, PIR, education, smoking, drinking, PA, BMI, and lipid-lowering drug use, except for the stratified variable itself. Interaction tests were performed for all the subgroups using a likelihood ratio test.

No imputations were performed. Statistical analyses were performed using R 4.3.2 and Free Statistics software version 1.9.2, employing the R package ‘survey’ version 4.2-1 for weighted analysis. Statistical *P* value was defined as a two-sided *P*-value < 0.05.

## Results

### Weighted attributes of the survey respondents from NHANES 2011–2018

This study included 4727 participants. In the highest VAT tertile, the participants were characterized by older age, obesity, male sex, non-Hispanic whites, and no alcohol consumption. Conversely, the lowest VAT tertile was associated with a higher education level, non-smoking behavior, engagement in PA, and non-use of lipid-lowering drugs. As the VAT area increased, TC, TG, and LDL-C concentrations increased, whereas HDL-C concentrations decreased. Furthermore, the PIR showed no significant differences across the three VAT groups. Additional details are provided in Table 1. The demographic characteristics of the included and excluded participants are shown in Supplementary Table 1.

**Table 1:** Weighted characteristics of participants in NHANES 2011–2018 according to tertiles of VAT area

		VAT area (cm^2^)			
Variable	Total	T1 (5.56–66.94)	T2 (66.95-117.91)	T3 (117.92–415.60)	*P* value
Number	4727	1576	1575	1576	
Age (years)	38.78(0.26)	31.97(0.45)	38.78(0.39)	45.11(0.31)	< 0.001
BMI (kg/m^2^)	28.87(0.16)	23.45(0.13)	28.72(0.18)	34.04(0.24)	< 0.001
Sex, n (%)					< 0.001
Female	2366(49.02)	868(55.41)	742(45.66)	756(46.17)	
Male	2361(50.98)	708(44.59)	833(54.34)	820(53.83)	
Ethnicity, n (%)					< 0.001
Non-Hispanic White	1760(63.34)	558(62.12)	520(59.12)	682(68.34)	
Non-Hispanic Black	973(11.45)	402(15.57)	329(11.68)	242(7.41)	
Mexican American	676(9.38)	127(5.49)	240(10.91)	309(11.60)	
Other Hispanic	479(6.75)	140(6.57)	167(7.55)	172(6.19)	
Other Race - Including Multi-Racial	839(9.07)	349(10.25)	319(10.74)	171(6.45)	
Poverty-income ratio, n (%)					0.54
≤1.3	1590(24.63)	538(26.06)	511(24.97)	541(22.99)	
>1.3,≤3.5	1724(35.70)	560(33.86)	587(36.32)	577(36.82)	
>3.5	1413(39.68)	478(40.08)	477(38.71)	458(40.19)	
Education, n (%)					0.03
Less than high school	846(13.53)	237(10.95)	279(14.15)	330(15.37)	
High school	1060(22.30)	354(21.87)	359(22.96)	347(22.10)	
More than high school	2821(64.17)	985(67.19)	937(62.89)	899(62.53)	
Smoking, n (%)					< 0.001
Never	2903(59.55)	1065(64.31)	972(60.34)	866(54.40)	
Former	810(19.88)	184(15.02)	266(18.60)	360(25.57)	
Now	1014(20.57)	327(20.67)	337(21.06)	350(20.03)	
Drinking, n (%)					0.04
No	3916(79.50)	1275(76.93)	1314(78.77)	1327(82.55)	
Yes	811(20.50)	301(23.07)	261(21.23)	249(17.45)	
Physical activity, n (%)					< 0.001
No	2077(41.27)	534(30.96)	672(38.64)	871(53.26)	
Yes	2650(58.73)	1042(69.04)	903(61.36)	705(46.74)	
Lowering lipid drug, n (%)					< 0.001
No	4314(90.32)	1543(97.62)	1458(91.97)	1313(82.03)	
Yes	413(9.68)	33(2.38)	117(8.03)	263(17.97)	
Total cholesterol (mg/dl)	185.00(161.00-213.00)	172.00(152.00-196.00)	190.00(165.00-215.00)	195.00(170.00-223.00)	< 0.001
Triglyceride (mg/dl)	92.00(62.00-137.00)	64.00(46.00–91.00)	92.00(66.00-133.00)	124.00(89.00-178.00)	< 0.001
HDL (mg/dl)	51.00(42.00–61.00)	59.00(49.00–69.00)	50.00(42.00–60.00)	46.00(39.00,54.00)	< 0.001
LDL (mg/dl)	111.00(89.00-135.00)	97.00(80.00-118.00)	116.00(94.00-138.00)	118.00(97.00-143.00)	< 0.001

Abbreviation: NHANES: National Health and Nutrition Examination Survey; T: tertile; BMI: body mass index; HDL: high-density lipoprotein; LDL: low-density lipoprotein

### Association between VAT and RC concentration among US adults in NHANES 2011–2018

In the univariable analysis, the VAT area exhibited a positive association with RC (β = 0.09, 95% confidence interval [CI]: 0.08 ~ 0.11). Compared with the first tertile, the second and third tertiles of VAT also showed positive associations with RC. Detailed information and associations between other variables and RC are provided in Supplementary Table 2. In the multivariate regression analyses, consistent positive associations between VAT and RC were observed across all three models. For every 1 cm^2^ increase in VAT, the β coefficients for RC were 0.09 (95%CI: 0.07 ~ 010), 0.09 (95%CI: 0.07 ~ 0.10), and 0.09 (95%CI: 0.08 ~ 0.11) in models 1, 2, and 3, respectively. Compared with those in the first VAT tertile, the β coefficients for RC in the second and third tertiles of VAT were 5.62 (95%CI: 4.60 ~ 6.65) and 12.08 (95%CI: 10.78 ~ 13.38) in model 1; 5.61 (95%CI: 4.60 ~ 6.62) and 12.04 (95%CI:10.73 ~ 13.35) in model 2; and 5.35 (95%CI: 4.08 ~ 6.63) and 11.49 (95%CI: 9.81 ~ 13.17) in model 3. All *P*-values for the trends were < 0.001 (Table 2).

**Table 2:** Association between VAT and RC in multivariable linear regression models among NHANES 2011–2018 adults

Variable	Model1		Model2		Model3	
	β(95%CI)	*P* value	β(95%CI)	*P* value	β(95%CI)	*P* value
VAT (per 1cm^2^)	0.09(0.07, 0.10)	< 0.001	0.09(0.07, 0.10)	< 0.001	0.09(0.08, 0.11)	< 0.001
VAT tertiles						
T1(5.56–66.94)	Ref (0)		Ref (0)		Ref (0)	
T2(66.95-117.91)	5.62(4.60, 6.65)	< 0.001	5.61(4.60, 6.62)	< 0.001	5.35(4.08, 6.63)	< 0.001
T3(117.92–415.60)	12.08(10.78,13.38)	< 0.001	12.04(10.73,13.35)	< 0.001	11.49(9.81,13.17)	< 0.001
*P* for trend		< 0.001		< 0.001		< 0.001

Abbreviation: NHANES: National Health and Nutrition Examination Surveys; RC: remnant cholesterol; VAT: visceral adipose tissue; CI: confidence interval; Ref: reference; BMI: body mass index

Model 1: adjusted for age, sex, and ethnicity

Model 2: adjusted for model 1 + education, poverty-income ratio, smoking, drinking, and physical activity

Model 3: adjusted for model 2 + BMI, and lowering lipid drug

### Nonlinear association between VAT and RC concentration among US adults in NHANES 2011–2018

**Fig. 2:**
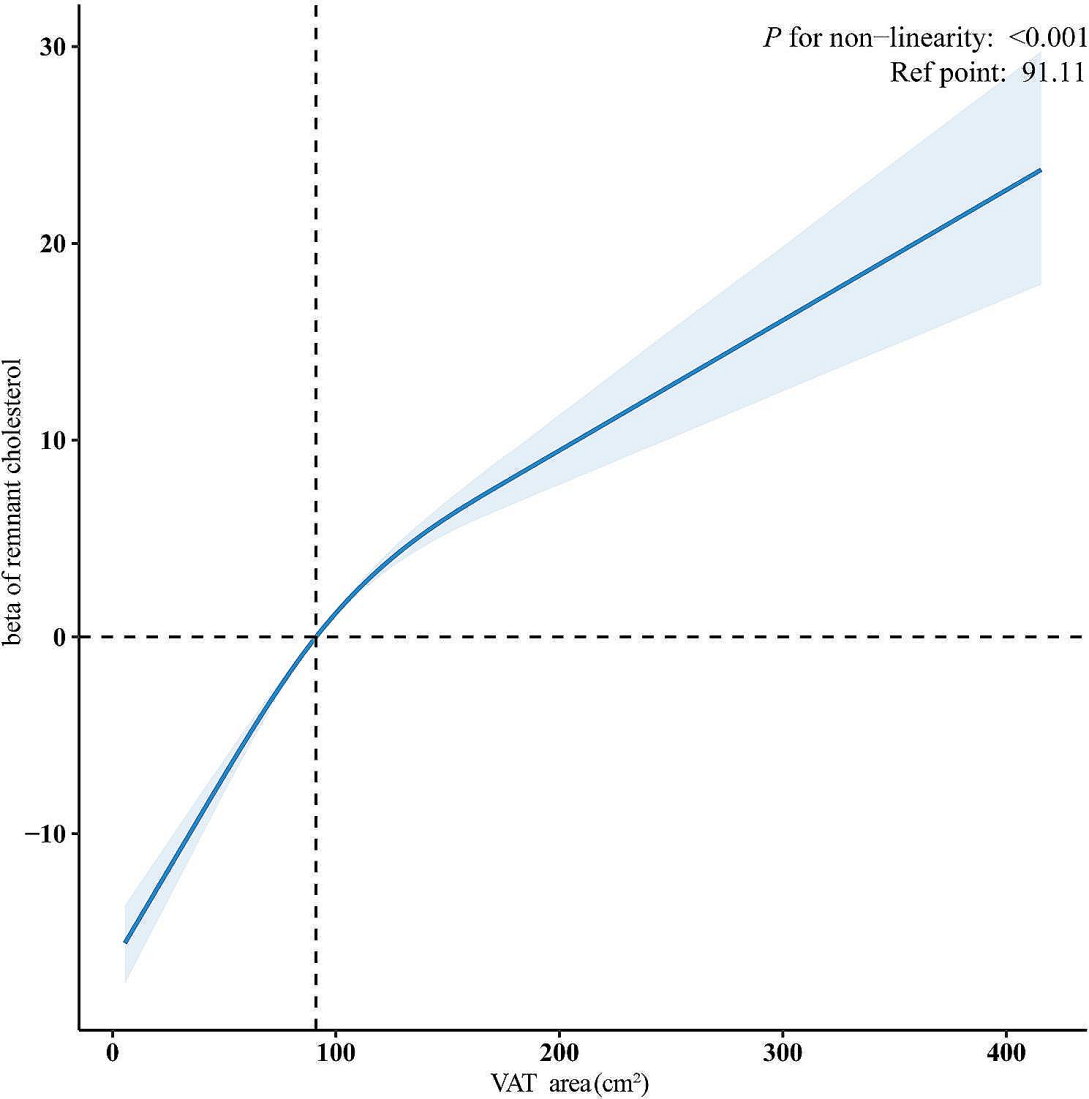
Restricted cubic spline analysis of the association between VAT area and RC in adults from NHANES 2011–2018 A restricted cubic spline with four knots at the 5th, 35th, 65th, and 95th percentiles of the VAT area The blue solid line and shaded areas represent the predicted value of β and 95% CIs of RC. The broken lines on the y-axis and x-axis represent the β coefficient of RC and the median of VAT. The model was adjusted for age, sex, ethnicity, education, poverty-income ratio, smoking, drinking, physical activity, BMI, and lowering lipid drugs Abbreviations: NHANES: National Health and Nutrition Examination Survey; RC: remnant cholesterol; VAT: visceral adipose tissue; BMI: body mass index; CI: confidence interval.

Using RCS analysis, a nonlinear association between VAT and RC was identified, with a *P*-value for the nonlinearity of < 0.001 (Fig. 2). In the crude two-piecewise regression models, the β coefficients for RC were 0.13 (95%CI:0.12 ~ 0.15) among individuals with VAT < 143 cm^2^ and 0.02 (95%CI: -0.01 ~ 0.05) among those with VAT ≥ 143 cm^2^. The adjusted two-piecewise regression models showed β coefficients of 0.13 (95%CI: 0.11 ~ 0.16) for RC among individuals with VAT < 143 cm^2^ and 0.02 (95%CI: -0.01 ~ 0.06) among those with VAT ≥ 143 cm^2^. Detailed findings are presented in Table 3.

**Table 3:** Association between VAT and RC using two-piecewise regression models in adults from NHANES 2011–2018

	Unadjusted model		Adjusted model^*^	
VAT (cm^2^)	β(95%CI)	*P* value	β(95%CI)	*P* value
< 143	0.13(0.12,0.15)	< 0.001	0.13(0.11, 0.16)	< 0.001
≥ 143	0.02(-0.01,0.05)	0.21	0.02(-0.01, 0.06)	0.15

Abbreviation: RC: remnant cholesterol; VAT: visceral adipose tissue; CI: confidence interval; BMI: body mass index

*: Adjusted for age, sex, ethnicity education, poverty-income ratio, smoking, drinking,

physical activity, BMI, and lowering lipid drug

### Association between VAT and RC concentration in the subgroup analyses among US adults from NHANES 2011–2018

A positive association was observed between VAT and RC across all subgroups. The β coefficients for RC were 0.10 (95%CI: 0.08 ~ 0.13) in those aged < 40 years and 0.09 (95%CI: 0.07 ~ 0.11) in those aged ≥ 40 years, with a *P*-value of 0.18 for interaction. Considering sex, the β coefficients for RC were 0.10 (95%CI: 0.08 ~ 0.11) in females and 0.09 (95%CI: 0.07 ~ 0.11) in males, with a *P*-value of 0.45 for interaction. Among individuals stratified by BMI, the β coefficients for RC were 0.14 (95%CI: 0.12 ~ 0.16) in those with BMI < 30 kg/m^2^ and 0.05 (95%CI: 0.04 ~ 0.07) in those with BMI ≥ 30 kg/m^2^, with a *P*-value of < 0.001 for interaction. Furthermore, the β coefficients for RC exhibited variability across ethnic groups, with values of 0.09 (95%CI: 0.07 ~ 0.12) for non-Hispanic White, 0.07 (95%CI: 0.05 ~ 0.09) for non-Hispanic Black, and 0.08 (95%CI: 0.05,0.12) for Mexican American, with a corresponding *P*-value of 0.10 for interaction. Additional details are presented in Supplementary Table 3.

## Discussion

In the current cross-sectional study of US adults aged 18–59 years, a positive nonlinear association between the VAT area and RC concentration was detected. Specifically, the strength of the association was pronounced when VAT levels were below 143 cm^2^, although this association weakened significantly for VAT levels ≥ 143 cm^2^, losing statistical significance. Notably, this positive association was more prominent among individuals with BMI < 30 kg/m^2^. The findings of this study offer a new perspective for understanding the nonlinear association between VAT and RC and may provide important reference points for clinical practice, aiding in the improvement of individual health management and preventive measures.

The findings of this study are consistent with those of a study conducted in China involving 5959 children aged 6–12 years [29], which had reported an association between RC and abdominal obesity as defined by the waist-to-height ratio; however, the authors did not explore the nonlinear nature of this association and limited the subgroup analysis to different living areas. In the current study, a positive association between abdominal fat and RC was observed in adults, and DXA was used to define the independent variables. Moreover, the current study identified a threshold saturation effect between the VAT and RC and conducted a more comprehensive subgroup analysis.

The link between RC and fat deposition in abdominal organs has been extensively explored. For instance, a Chinese cohort study followed 16,173 non-obese participants with BMI < 25 kg/m^2^ and found an association between RC and non-alcoholic fatty liver disease after a 5-year follow-up [30]. Similarly, a positive association between the VAT area measured using DXA and RC was observed within an indicative subset of the general American grown-up demographic, regardless of obesity status. In another study involving 348 participants undergoing abdominal magnetic resonance imaging [31], an association between RC and total intrapancreatic fat deposition was detected in a fully adjusted model without conducting subgroup analysis. Likewise, the current study focused on VAT as an independent variable rather than being limited to a specific abdominal organ. Moreover, the study included a larger participant sample and utilized weighted methods to enhance the applicability of the results when compared with the aforementioned study. Additionally, a separate Mendelian randomization analysis has explored the causal links between RC and cardiometabolic disease risk factors [32] and identified no genetic link between RC and body fat; the authors did not analyze the genetic association between VAT and RC or investigate nonlinear associations in Mendelian randomization studies.

Elevated VAT levels can induce fat dysfunction and chronic local inflammation, which is characterized by the infiltration of M1 macrophages that produce reactive oxygen free radicals and cytokines involved in inflammation, including tumor necrosis factor-alpha and interleukins 6 [33, 34]. This persistent low-level inflammation contributes to the emergence of dyslipidemia [35]. It is noteworthy that this association between low-grade inflammation and dyslipidemia may be bidirectional, indicating that chronic subclinical inflammation may trigger dyslipidemia and vice versa. Adipokines, namely adiponectin and leptin, are hormones secreted by adipose tissue that regulate systemic metabolism and inflammation. Clinical studies have demonstrated an association between hypoadiponectinemia and dyslipidemia [36]. Although these studies have previously reported a positive association between VAT and dyslipidemia, the current study further indicates that this association may not be linear. To enhance the understanding of the association between VAT and RC, an intervention study aimed at altering the VAT and examining its impact on RC would be ideal.

In the current study, the observed nonlinear association between VAT and RC could be attributed to several factors, including the saturation of metabolic regulation, threshold effect of inflammatory responses, and heterogeneity of adipose tissue function. However, further studies are required to elucidate these mechanisms.

### Study strengths and limitations

This study has several strengths. First, the inclusion of a diverse participant pool, in combination with the use of weighted methods, ensured that the findings accurately reflected the broader population of US adults aged 18–59 years. Second, the RCS analysis uncovered a nonlinear association between VAT and RC, indicating a positive association between VAT and RC, particularly when VAT levels were < 143cm^2^. Finally, the study employed a comprehensive multivariate analysis with full adjustment for covariates. Subgroup evaluations were also performed to enhance the credibility of the outcomes.

Nevertheless, this study has several limitations. First, its cross-sectional nature precludes the establishment of causality. Second, as the upper age limit for participants undergoing DXA examinations in the NHANES was 59 years, the results can only be generalized to US adults within this age range. Third, although the Friedewald formula was used to calculate LDL-C concentrations, potential discrepancies from the actual LDL-C concentrations may have arisen. Unfortunately, the NHANES dataset does not include directly measured LDL-C concentrations. Finally, by incorporating sampling weights, the findings of this study are representative of the broader population of US adults aged 18–59 years. However, the generalizability of this nonlinear association to other populations remains unclear. Accordingly, additional investigations involving diverse populations are required to address these limitations.

## Conclusions

A positive association was observed between the VAT area and RC concentration in adults aged 18–59 years, particularly when the VAT area was < 143 cm^2^. These findings suggest that reducing the VAT area could be advantageous for decreasing the RC concentration, potentially lowering the risk of cardiovascular disease, especially among individuals with a BMI < 30 kg/m^2^. Routine VAT measurement can serve as an early indicator of increased cardiovascular risk. This can help clinicians to identify high-risk patients earlier and promptly initiate preventive measures. For patients with higher VAT areas, clinicians can recommend targeted lifestyle modifications, such as specific dietary adjustments and tailored exercise programs aimed at reducing VAT, thereby potentially lowering RC levels and cardiovascular risk. Furthermore, longitudinal studies or interventional trials are required to confirm the observed associations and to elucidate causality.

### Electronic supplementary material

Below is the link to the electronic supplementary material.

Supplementary Material 1

Supplementary Material 2

Supplementary Material 3

## Data Availability

The datasets generated and analyzed during the current study are available in the NHANES repository, https://www.cdc.gov/nchs/nhanes/.

## Abbreviations

VAT: visceral adipose tissue
RC: remnant cholesterol
TRLs: Triglyceride-rich lipoproteins
VLDL: very low-density lipoprotein
IDL: intermediate-density lipoprotein
NHANES: National Health and Nutrition Examination Survey
LDL-C: low-density lipoprotein cholesterol
BMI: body mass index
PIR: poverty-income ratio
NCHS: National Center for Health Statistics
MEC: mobile examination center
TC: Total cholesterol
TG: triglyceride
HDL-C: high-density lipoprotein cholesterol
DXA: dual-energy X-ray absorptiometry
PA: physical activity
IQR: interquartile range
RCS: restricted cubic spline
CI: confidence interval
