# Supplementary material for: Nonlinear association between visceral adipose tissue area and remnant cholesterol in US adults: a cross-sectional study
Source: Lipids Health Dis. 2024 Jul 25;23:228. doi: 10.1186/s12944-024-02211-z (PMC11270912; doi:10.1186/s12944-024-02211-z)
Supplement: Supplementary file 1 — Supplementary Material 1 [file 12944_2024_2211_MOESM1_ESM.docx]

**Table S1**. Comparison of the characteristics between the excluded subjects and those included in NHANES 2011-2018.

| Variable | Total | Included | Excluded | *P* value |
| --- | --- | --- | --- | --- |
| Number | 12946 | 4727 | 8219 |  |
| Age, years | 38.70(0.24) | 39.21(0.27) | 38.39(0.29) | 0.01 |
| BMI, kg/m^2^ | 28.86(0.13) | 28.91(0.16) | 28.82(0.16) | 0.63 |
| VAT, cm^2^ | 103.34(1.20) | 105.08(1.44) | 102.24(1.42) | 0.08 |
| Total cholesterol, mg/dl | 187.00(162.00,214.00) | 186.00(161.00,213.00) | 188.00(163.00,215.00) | 0.08 |
| Triglyceride, mg/dl | 94.00(63.00,141.00) | 93.00(62.00,138.00) | 100.00(64.00,166.00) | 0.01 |
| HDL, mg/dl | 50.00(41.00,61.00) | 51.00(42.00,61.00) | 50.00(41.00,61.00) | < 0.001 |
| LDL, mg/dl | 110.00(89.00,134.00) | 111.00(89.00,135.00) | 108.00(89.00,128.00) | 0.04 |
| Sex, n (%) |  |  |  | 0.43 |
| Female | 6365(48.22) | 2366(48.70) | 3999(47.91) |  |
| Male | 6581(51.78) | 2361(51.30) | 4220(52.09) |  |
| Ethnicity, n (%) |  |  |  | < 0.001 |
| Non-Hispanic White | 4417(61.26) | 1760(64.27) | 2657(59.36) |  |
| Non-Hispanic Black | 2910(11.90) | 973(10.34) | 1937(12.87) |  |
| Mexican American | 1902(10.13) | 676(9.53) | 1226(10.51) |  |
| Other Hispanic | 1318(7.19) | 479(6.85) | 839(7.41) |  |
| Other Race - Including Multi-Racial | 2399(9.52) | 839(9.01) | 1560(9.85) |  |
| Poverty-income ratio, n (%) |  |  |  | 0.33 |
| ≤1.3 | 4080(22.65) | 1590(24.05) | 2490(24.55) |  |
| >1.3, ≤3.5 | 4194(31.92) | 1724(35.50) | 2470(33.45) |  |
| >3.5 | 3571(38.48) | 1413(40.44) | 2158(42.00) |  |
| Education, n (%) |  |  |  | 0.36 |
| Less than high school | 2525(13.92) | 846(13.29) | 1679(14.31) |  |
| High school | 3013(22.56) | 1060(22.07) | 1953(22.86) |  |
| More than high school | 7406(63.52) | 2821(64.63) | 4585(62.83) |  |
| Smoking, n (%) |  |  |  | 0.17 |
| Never | 7866(58.65) | 2903(59.23) | 4963(59.45) |  |
| Former | 2036(19.12) | 810(20.33) | 1226(18.72) |  |
| Now | 2787(21.03) | 1014(20.44) | 1773(21.84) |  |
| Drinking, n (%) |  |  |  | 0.24 |
| No | 10092(75.21) | 3916(79.37) | 6176(78.01) |  |
| Yes | 2175(20.53) | 811(20.63) | 1364(21.99) |  |
| Physical activity, n (%) |  |  |  | 0.47 |
| No | 5716(40.58) | 2077(41.18) | 3639(40.20) |  |
| Yes | 7230(59.42) | 2650(58.82) | 4580(59.80) |  |
| Lowering lipid drug, n (%) |  |  |  | 0.002 |
| No | 11911(91.18) | 4314(89.92) | 7597(92.06) |  |
| Yes | 1028(8.76) | 413(10.08) | 615(7.94) |  |

Abbreviation: NHANES, National Health and Nutrition Examination Survey; BMI, body mass index;

HDL, high-density lipoprotein; LDL, low-density lipoprotein; VAT, visceral adipose tissue.

Excluded participants: 1. Those with missing data on LDL (n=7244). 2. Those with missing data on BMI, poverty-income

ratio, smoking, drinking, and lipid-lowering drugs (n=813). 3. Those had a zero value of 2-year fasting lipid weight (n=162).

**Table S2.** Association between univariable and RC concentration in adults

from NHANES 2011-2018.

|  | β(95% CI) | *P* value |
| --- | --- | --- |
| VAT, cm^2^ | 0.09(0.08,0.11) | <0.0001 |
| VAT, tertiles |  |  |
| T1 | ref |  |
| T2 | 6.49(5.49, 7.48) | <0.001 |
| T3 | 13.42(12.24,14.61) | <0.001 |
| Age, years | 0.23(0.19,0.27) | <0.001 |
| BMI, kg/m^2^ | 0.45(0.38,0.53) | <0.001 |
| Sex |  |  |
| Female | ref |  |
| Male | 4.16(3.25,5.07) | <0.001 |
| Ethnicity |  |  |
| Non-Hispanic White | ref |  |
| Non-Hispanic Black | -5.78(-6.90,-4.66) | <0.001 |
| Mexican American | 0.59(-0.66, 1.84) | 0.35 |
| Other Hispanic | -0.85(-2.45, 0.75) | 0.29 |
| Other Race - Including Multi-Racial | -0.39(-1.85, 1.06) | 0.59 |
| Poverty-income ratio |  |  |
| ≤1.3 | ref |  |
| >1.3,≤3.5 | 0.08(-1.16,1.31) | 0.90 |
| >3.5 | 0.16(-0.98,1.30) | 0.78 |
| Education |  |  |
| Less than high school | ref |  |
| High school | -0.82(-2.71, 1.07) | 0.39 |
| More than high school | -1.47(-2.62, -0.32) | 0.01 |
| Smoking |  |  |
| never | ref |  |
| former | 3(1.34,4.66) | <0.001 |
| now | 3.8(2.36,5.23) | <0.001 |
| Drinking |  |  |
| No | ref |  |
| Yes | 0.18(-1.29,1.66) | 0.80 |
| Physical activity |  |  |
| No | ref |  |
| Yes | -2.8(-3.92, -1.68) | <0.001 |
| Lowering lipid drug |  |  |
| No | ref |  |
| Yes | 5.77(3.55,8.00) | <0.001 |

Abbreviation: NHANES, National Health and Nutrition Examination Survey; T, tertile; BMI, body mass index; CI, confidence interval; ref, reference; RC, remnant cholesterol; VAT, visceral adipose tissue.

**Table S3.** Subgroup analysis of the association between VAT and RC concentration

in adults from NHANES 2011-2018.

| Subgroup | Number | β (95%CI) | *P* for interaction |
| --- | --- | --- | --- |
| Age, years |  |  | 0.18 |
| <40 | 2459 | 0.10( 0.08, 0.13) |  |
| ≥40 | 2268 | 0.09( 0.07, 0.11) |  |
| Sex |  |  | 0.45 |
| Female | 2366 | 0.10( 0.08, 0.11) |  |
| Male | 2361 | 0.09( 0.07, 0.11) |  |
| BMI, kg/m^2^ |  |  | <0.001 |
| <30 | 3001 | 0.14(0.12,0.16) |  |
| ≥30 | 1726 | 0.05(0.04,0.07) |  |
| Ethnicity |  |  | 0.10 |
| Non-Hispanic White | 1760 | 0.09( 0.07,0.12) |  |
| Non-Hispanic Black | 973 | 0.07( 0.05, 0.09) |  |
| Mexican American | 676 | 0.08( 0.05,0.12) |  |
| Other Hispanic | 479 | 0.09( 0.05,0.13) |  |
| Other Race - Including Multi-Racial | 839 | 0.12( 0.07,0.17) |  |

Abbreviation: NHANES, National Health and Nutrition Examination Survey; BMI, body mass index; CI, confidence interval; RC, remnant cholesterol; VAT, visceral adipose tissue.

Each model in the subgroups was adjusted for age, sex, ethnicity, education, poverty-income ratio, smoking, drinking, physical activity, BMI, and lowering lipid drugs, except for the stratified variable itself.
