# Supplementary material for: Nonlinear association between visceral adipose tissue area and remnant cholesterol in US adults: a cross-sectional study
Source: Lipids Health Dis. 2024 Jul 25;23:228. doi: 10.1186/s12944-024-02211-z (PMC11270912; doi:10.1186/s12944-024-02211-z)
Supplement: Supplementary file 3 — Supplementary Material 3 [file 12944_2024_2211_MOESM3_ESM.pdf]

20240709220105100817310766638284

WORD COUNT 4140
TIME SUBMITTED 09-JUL-2024 10:02PM
PAPER ID 110160109

Abstract

Background: Excessive visceral adipose tissue (VAT) is associated with a spectrum of diseases, including diabetes, cancer, and cardiovascular diseases. Remnant cholesterol (RC), denoting cholesterol within triglyceride-rich lipoproteins and their metabolic byproducts, has been identified as a key contributor to cardiovascular diseases and related mortality. However, the association between the VAT and RC remains unclear. In this study, we aimed to provide new evidence regarding the association between VAT and RC concentrations.

Methods: 4727 individuals aged 18–59 were selected from the National Health and Nutrition Examination Survey conducted between 2011 and 2018 as study participants. This study utilized several weighted linear regression models and a restricted cubic spline (RCS) to explore the association and potential nonlinearities between VAT and RC. Subgroup analyses were performed to determine the consistency of findings.

Results: The mean VAT value was 103.82±1.42 cm^2, and the median RC value was 18 mg/dl. VAT demonstrated a positive association with RC in a fully adjusted model, with a β and 95% confidence interval (CI) of 0.10 (0.08, 0.12) after adjustment for potential confounders. Analysis using RCS revealed a nonlinear association between the VAT area and RC ($P < 0.001$ for nonlinearity). Adjusted two-piecewise regression models demonstrated β coefficients of 0.13 (95%CI: 0.11~0.16, $P < 0.001$) for RC in individuals with VAT < 143 cm^2, and 0.02 (95%CI: -0.01~0.06, $P = 0.15$) for those with VAT ≥ 143 cm^2. Interactions were observed among the body mass index (BMI)

subgroup; the β coefficients for RC were 0.14 (95%CI: 0.12~0.16) in those with BMI <30 kg/m^2 and 0.05 (95%CI:0.04~0.07) in those with BMI ≥30 kg/m^2, with a *P*-value of <0.001 for interaction.

Conclusions: This study identified a nonlinear association between VAT and RC in American adults. Reducing the VAT area may be beneficial in lowering RC concentration, particularly when VAT is <143 cm^2 and those with a BMI <30 kg/m^2.

Keywords: Remnant cholesterol; Visceral adipose tissue; Nonlinear association; National Health and Nutrition Examination Survey.

1. Background

Extensive research has established that adipose tissue not only functions as an energy storage site but also as a notable endocrine organ[1]. Excess adipose tissue, particularly visceral adipose tissue (VAT), is implicated in numerous obesity-related disease processes[2]. VAT has been linked to decreased insulin efficiency[3], non-insulin-dependent diabetes[4], as well as their related complications such as diabetic nephropathy and retinopathy[5, 6]. Additionally, excess VAT is associated with a greater probability of developing cancer and a poorer prognosis for colorectal and liver cancers[7, 8]. Importantly, VAT is also associated with a higher prevalence of cardiovascular diseases[9], which are identified as primary contributors to mortality[10].

Very low-density lipoproteins (VLDL), intermediate-density lipoproteins (IDL), and chylomicron remnants jointly form triglyceride-rich lipoproteins (TRLs)[11]. The cholesterol present in TRLs and the products of their metabolism are referred to as remnant cholesterol (RC)[12], which serves as the primary source of lipid-dependent residual risk in cardiovascular diseases [13, 14]. An elevated RC concentration is associated with higher cardiovascular disease mortality[15, 16]. Furthermore, recent studies have revealed additional associations between RC and the presence of non-alcoholic fatty liver disease[17], higher long-term mortality rates in individuals with metabolic dysfunction-associated fatty liver disease[18], new-onset prediabetes[19], and hip bone mineral density[20].

Both VAT and RC play crucial roles as risk factors for the development of

cardiovascular diseases; however, their association has not been extensively studied. To address this knowledge gap and examine the hypothesized positive link between the VAT area and RC concentration, a cross-sectional study was carried out, using data from the National Health and Nutrition Examination Survey (NHANES). Unlike previous studies that primarily focused on the individual effects of VAT and RC on cardiovascular health, we examined the direct association between VAT and RC. Additionally, the use of the curve-fitting method facilitated a detailed exploration of potential nonlinear associations, providing new insights into the association between VAT and RC, which remains poorly clarified.

2. Methods

2.1 Research subjects

During the initial phase, 68897 participants aged 18 years were enrolled from the NHANES 2011–2018 dataset. Subsequently, individuals lacking VAT area data and those with missing information on low-density lipoprotein cholesterol (LDL-C) concentrations, poverty-income ratio (PIR), smoking habits, alcohol intake, lipid-lowering drug use, or body mass index (BMI) were excluded. Additionally, individuals with a fasting lipid 2-year weight of zero were excluded from the analysis. Ultimately, the study population comprised 4727 participants (Figure 1). The NHANES program was conducted by the National Center for Health Statistics (NCHS) and approved by the NCHS Ethics Review Board. The guidelines specified in the Strengthening the Reporting of Observational Studies in Epidemiology statement were strictly followed in this study[21].

2.2 Measurement of RC

Serum samples collected from the NHANES mobile examination center (MEC) were processed, stored, and forwarded to the University of Minnesota, Minneapolis, for analysis. During the MEC visit, the participants were queried regarding their fasting status. Blood samples were collected from individuals who met the 9-h fasting requirement for lipid level assessment. Enzymatic or immunological methods were used to measure the concentrations of total cholesterol (TC), triglycerides (TG), and high-density lipoprotein cholesterol (HDL-C)[22]. The Friedewald formula was applied to calculate LDL-C[23], with TG values of ≤400 mg/dl; when TG exceeded this threshold, LDL-C data were considered missing[22]. Finally, the RC was determined by deducting the combined values of LDL-C and HDL-C from the TC[24].

2.3 Measurement of VAT area

The NHANES conducted whole-body dual-energy X-ray absorptiometry (DXA) scans on participants aged 8–59 years who met eligibility requirements. Participants were excluded from the DXA scan if they were pregnant, had used radiographic contrast material (such as barium) within the previous week, weighed more than 450 lb, or were >6 feet and 5 inches in height. Scans were performed at the MEC, with VAT defined using the analysis provided by the Hologic APEX software (version 4.0).[25]. The VAT area, which identified the fat within the abdomen, was evaluated between the fourth and fifth lumbar vertebrae. DXA examinations were performed by radiology technologists with proper training and certification[26].

2.4 Covariates

The demographic information used as covariates included age, sex (male or female), and ethnic background (Mexican American, non-Hispanic White, non-Hispanic Black, other Hispanic, and other/multi-racial groups). Additionally, the PIR was divided into low-income (≤1.3), middle-income (1.3 to 3.5), and high-income (>3.5). Educational level was classified as less than high school, high school diploma, and education beyond high school. Lifestyle factors consisted of smoking categories (never, former, current), alcohol consumption (≥2 daily drinks for males, ≥1 daily drink for females), and physical activity (PA) levels (determined by participation in recreational activities). Body measurements were determined using BMI, which was calculated by dividing weight (kg) by height squared (m^2). The use of medications to lower lipid levels, including β-hydroxy β-methylglutaryl-CoA reductase inhibitors, cholestyramine, colesevelam, ezetimibe, fenofibrate, gemfibrozil, and niacin, were also considered.

2.5 Statistical analysis

Between December 2023 and May 2024, analyses were performed using NHANES guidelines by considering the intricate sampling design and applying the appropriate sampling weights. Specifically, the sampling weight was equal to one-quarter of the two-year fasting lipid weight. Weighted participant attributes are expressed as mean (standard error) for normally distributed continuous variables and as median (interquartile range [IQR]) for distributions with skewness, whereas categorical data are presented as unweighted numbers and weighted percentages. Differences among

VAT tertiles were assessed using $\chi 2$ for categorical variables, one-way analysis of variance for normally distributed data, and the Kruskal-Wallis H test for skewed distributions.

Univariate and multivariate linear regression analyses were performed to explore the association between the VAT and RC across the three models. Model 1 was adjusted for age, sex, and ethnicity, while Model 2 was additionally adjusted for educational level, cigarette consumption, alcohol consumption, PIR, and PA. In Model 3, further adjustments were made for BMI and the use of lipid-lowering drugs. Covariates were selected based on available literature[27, 28], clinical judgment, and associations in the univariate analysis ($P < 0.05$). To examine the association of VAT with RC, the VAT area was classified into tertiles and analyzed using multivariate linear regression models.

To account for nonlinear association, a restricted cubic spline (RCS) containing knots positioned at the 5th, 35th, 65th, and 95th distribution points of the exposure distribution in Model 3 was utilized. The likelihood ratio test was employed to evaluate the nonlinearity. If nonlinearity was detected, a two-piecewise linear regression model was constructed around the turning point.

Prespecified analyses of subgroups were performed depending on age (<40, ≥40), sex, ethnicity, and BMI (<30, ≥30) in the adjusted model considering age, sex, ethnicity, PIR, education, smoking, drinking, PA, BMI, and lipid-lowering drug use, except for the stratified variable itself. Interaction tests were performed for all the subgroups using a likelihood ratio test.

No imputations were performed. Statistical analyses were performed using R 4.3.2 and Free Statistics software version 1.9.2, employing the R package 'survey' version 4.2-1 for weighted analysis. Statistical P value was defined as a two-sided P-value < 0.05.

3. Results

3.1 Weighted attributes of the survey respondents from NHANES 2011–2018.

This study included 4727 participants. In the highest VAT tertile, the participants were characterized by older age, obesity, male sex, non-Hispanic whites, and no alcohol consumption. Conversely, the lowest VAT tertile was associated with a higher education level, non-smoking behavior, engagement in PA, and non-use of lipid-lowering drugs. As the VAT area increased, TC, TG, and LDL-C concentrations increased, whereas HDL-C concentrations decreased. Furthermore, the PIR showed no significant differences across the three VAT groups. Additional details are provided in Table 1. The demographic characteristics of the included and excluded participants are shown in Supplementary Table 1.

3.2 Association between VAT and RC concentration among US adults in NHANES 2011–2018.

In the univariable analysis, the VAT area exhibited a positive association with RC (β=0.09, 95% confidence interval [CI]: 0.08~0.11). Compared with the first tertile, the second and third tertiles of VAT also showed positive associations with RC. Detailed information and associations between other variables and RC are provided in Supplementary Table 2. In the multivariate regression analyses, consistent positive

associations between VAT and RC were observed across all three models. For every 1 cm^2 increase in VAT, the β coefficients for RC were 0.09 (95%CI: 0.07~010), 0.09 (95%CI: 0.07~0.10), and 0.09 (95%CI: 0.08~0.11) in models 1, 2, and 3, respectively. Compared with those in the first VAT tertile, the β coefficients for RC in the second and third tertiles of VAT were 5.62 (95%CI: 4.60~6.65) and 12.08 (95%CI: 10.78~13.38) in model 1; 5.61 (95%CI: 4.60~6.62) and 12.04 (95%CI:10.73~13.35) in model 2; and 5.35 (95%CI: 4.08~6.63) and 11.49 (95%CI: 9.81~13.17) in model 3. All *P*-values for the trends were <0.001 (Table 2).

3.3 Nonlinear association between VAT and RC concentration among US adults in NHANES 2011–2018.

Using RCS analysis, a nonlinear association between VAT and RC was identified, with a *P*-value for the nonlinearity of <0.001 (Figure 2). In the crude two-piecewise regression models, the β coefficients for RC were 0.13 (95%CI:0.12~0.15) among individuals with VAT <143 cm^2 and 0.02 (95%CI: -0.01~0.05) among those with VAT ≥143 cm^2. The adjusted two-piecewise regression models showed β coefficients of 0.13 (95%CI: 0.11~0.16) for RC among individuals with VAT <143 cm^2 and 0.02 (95%CI: -0.01~0.06) among those with VAT ≥143 cm^2. Detailed findings are presented in Table 3.

3.4 Association between VAT and RC concentration in the subgroup analyses among US adults from NHANES 2011–2018.

A positive association was observed between VAT and RC across all subgroups. The β coefficients for RC were 0.10 (95%CI: 0.08~0.13) in those aged <40 years and 0.09

(95%CI: 0.07~0.11) in those aged ≥40 years, with a *P*-value of 0.18 for interaction. Considering sex, the β coefficients for RC were 0.10 (95%CI: 0.08~0.11) in females and 0.09 (95%CI: 0.07~0.11) in males, with a *P*-value of 0.45 for interaction. Among individuals stratified by BMI, the β coefficients for RC were 0.14 (95%CI: 0.12~0.16) in those with BMI <30 kg/m^2 and 0.05 (95%CI: 0.04~0.07) in those with BMI ≥30 kg/m^2, with a *P*-value of <0.001 for interaction. Furthermore, the β coefficients for RC exhibited variability across ethnic groups, with values of 0.09 (95%CI: 0.07~0.12) for non-Hispanic White, 0.07 (95%CI: 0.05~0.09) for non-Hispanic Black, and 0.08 (95%CI: 0.05,0.12) for Mexican American, with a corresponding *P*-value of 0.10 for interaction. Additional details are presented in Supplementary Table 3.

4. Discussion

In the current cross-sectional study of US adults aged 18–59 years, a positive nonlinear association between the VAT area and RC concentration was detected. Specifically, the strength of the association was pronounced when VAT levels were below 143 cm^2, although this association weakened significantly for VAT levels ≥143 cm^2, losing statistical significance. Notably, this positive association was more prominent among individuals with BMI <30 kg/m^2. The findings of this study offer a new perspective for understanding the nonlinear association between VAT and RC and may provide important reference points for clinical practice, aiding in the improvement of individual health management and preventive measures.

The findings of this study are consistent with those of a study conducted in China involving 5959 children aged 6–12 years [29], which had reported an association

between RC and abdominal obesity as defined by the waist-to-height ratio; however, the authors did not explore the nonlinear nature of this association and limited the subgroup analysis to different living areas. In the current study, a positive association between abdominal fat and RC was observed in adults, and DXA was used to define the independent variables. Moreover, the current study identified a threshold saturation effect between the VAT and RC and conducted a more comprehensive subgroup analysis.

The link between RC and fat deposition in abdominal organs has been extensively explored. For instance, a Chinese cohort study followed 16173 non-obese participants with BMI < 25 kg/m^2 and found an association between RC and non-alcoholic fatty liver disease after a 5-year follow-up[30]. Similarly, a positive association between the VAT area measured using DXA and RC was observed within an indicative subset of the general American grown-up demographic, regardless of obesity status. In another study involving 348 participants undergoing abdominal magnetic resonance imaging[31], an association between RC and total intrapancreatic fat deposition was detected in a fully adjusted model without conducting subgroup analysis. Likewise, the current study focused on VAT as an independent variable rather than being limited to a specific abdominal organ. Moreover, the study included a larger participant sample and utilized weighted methods to enhance the applicability of the results when compared with the aforementioned study. Additionally, a separate Mendelian randomization analysis has explored the causal links between RC and cardiometabolic disease risk factors[32] and identified no genetic link between RC and body fat; the

authors did not analyze the genetic association between VAT and RC or investigate nonlinear associations in Mendelian randomization studies.

Elevated VAT levels can induce fat dysfunction and chronic local inflammation, which is characterized by the infiltration of M1 macrophages that produce reactive oxygen free radicals and cytokines involved in inflammation, including tumor necrosis factor-alpha and interleukins 6[33, 34]. This persistent low-level inflammation contributes to the emergence of dyslipidemia [35]. It is noteworthy that this association between low-grade inflammation and dyslipidemia may be bidirectional, indicating that chronic subclinical inflammation may trigger dyslipidemia and vice versa. Adipokines, namely adiponectin and leptin, are hormones secreted by adipose tissue that regulate systemic metabolism and inflammation. Clinical studies have demonstrated an association between hypoadiponectinemia and dyslipidemia [36]. Although these studies have previously reported a positive association between VAT and dyslipidemia, the current study further indicates that this association may not be linear. To enhance the understanding of the association between VAT and RC, an intervention study aimed at altering the VAT and examining its impact on RC would be ideal.

In the current study, the observed nonlinear association between VAT and RC could be attributed to several factors, including the saturation of metabolic regulation, threshold effect of inflammatory responses, and heterogeneity of adipose tissue function. However, further studies are required to elucidate these mechanisms.

Study strengths and limitations

This study has several strengths. First, the inclusion of a diverse participant pool, in combination with the use of weighted methods, ensured that the findings accurately reflected the broader population of US adults aged 18–59 years. Second, the RCS analysis uncovered a nonlinear association between VAT and RC, indicating a positive association between VAT and RC, particularly when VAT levels were < 143cm^2. Finally, the study employed a comprehensive multivariate analysis with full adjustment for covariates. Subgroup evaluations were also performed to enhance the credibility of the outcomes.

Nevertheless, this study has several limitations. First, its cross-sectional nature precludes the establishment of causality. Second, as the upper age limit for participants undergoing DXA examinations in the NHANES was 59 years, the results can only be generalized to US adults within this age range. Third, although the Friedewald formula was used to calculate LDL-C concentrations, potential discrepancies from the actual LDL-C concentrations may have arisen. Unfortunately, the NHANES dataset does not include directly measured LDL-C concentrations. Finally, by incorporating sampling weights, the findings of this study are representative of the broader population of US adults aged 18–59 years. However, the generalizability of this nonlinear association to other populations remains unclear. Accordingly, additional investigations involving diverse populations are required to address these limitations.

5. Conclusions

A positive association was observed between the VAT area and RC concentration in

adults aged 18–59 years, particularly when the VAT area was < 143 cm^2. These findings suggest that reducing the VAT area could be advantageous for decreasing the RC concentration, potentially lowering the risk of cardiovascular disease, especially among individuals with a BMI <30 kg/m^2. Routine VAT measurement can serve as an early indicator of increased cardiovascular risk. This can help clinicians to identify high-risk patients earlier and promptly initiate preventive measures. For patients with higher VAT areas, clinicians can recommend targeted lifestyle modifications, such as specific dietary adjustments and tailored exercise programs aimed at reducing VAT, thereby potentially lowering RC levels and cardiovascular risk. Furthermore, longitudinal studies or interventional trials are required to confirm the observed associations and to elucidate causality.

Reference

1. Cypess AM: **Reassessing Human Adipose Tissue.** *N Engl J Med* 2022, **386:**768-779.

2. Zhang X, Ha S, Lau HC, Yu J: Excess body weight: Novel insights into its roles in obesity comorbidities. *Semin Cancer Biol* 2023, 92:16-27.

3. Iacobini C, Pugliese G, Blasetti Fantauzzi C, Federici M, Menini S: **Metabolically healthy versus metabolically unhealthy obesity.** *Metabolism* 2019, **92:**51-60.

4. Després JP: Intra-abdominal obesity: an untreated risk factor for Type 2 diabetes and cardiovascular disease. *J Endocrinol Invest* 2006, 29:77-82.

5. Tao M, Zhou G, Liu J, He M, Luo X, Wang C, Zhang L: Visceral adipose tissue and risk of diabetic nephropathy: A Mendelian randomization study. *Diabetes Res Clin Pract* 2024, 209:111586.

6. Chen J, Li YT, Niu Z, He Z, Xie YJ, Hernandez J, Huang W, Wang HHX: Association of Visceral Obesity Indices With Incident Diabetic Retinopathy in Patients With Diabetes: Prospective Cohort Study. *JMIR Public Health Surveill* 2024, 10:e48120.

7. Guiu B, Petit JM, Bonnetain F, Ladoire S, Guiu S, Cercueil JP, Krausé D, Hillon P, Borg C, Chauffert B, Ghiringhelli F: Visceral fat area is an independent predictive biomarker of outcome after first-line bevacizumab-based treatment in metastatic colorectal cancer. *Gut* 2010, 59:341-347.

8. Xu FQ, Xu QY, Zhu ZJ, Jin L, Ye TW, Du CF, Gao ZY, Huang XK, Zhang Z, Jin

LM, Yao WF: Visceral and ectopic fat are more predictively associated with primary liver cancer than overall obesity from genetic sights: A Mendelian randomization study. *Int J Cancer* 2024, 154:530-537.

9. Lotta LA, Wittemans LBL, Zuber V, Stewart ID, Sharp SJ, Luan J, Day FR, Li C, Bowker N, Cai L, et al: Association of Genetic Variants Related to Gluteofemoral vs Abdominal Fat Distribution With Type 2 Diabetes, Coronary Disease, and Cardiovascular Risk Factors. *Jama* 2018, 320:2553-2563.

10. Tsao CW, Aday AW, Almarzooq ZI, Alonso A, Beaton AZ, Bittencourt MS, Boehme AK, Buxton AE, Carson AP, Commodore-Mensah Y, et al: **Heart Disease and Stroke Statistics-2022 Update: A Report From the American Heart Association.** *Circulation* 2022, **145:**e153-e639.

11. Varbo A, Nordestgaard BG: Remnant Cholesterol and Triglyceride-Rich Lipoproteins in Atherosclerosis Progression and Cardiovascular Disease. *Arterioscler Thromb Vasc Biol* 2016, 36:2133-2135.

12. Varbo A, Benn M, Nordestgaard BG: Remnant cholesterol as a cause of ischemic heart disease: evidence, definition, measurement, atherogenicity, high risk patients, and present and future treatment. *Pharmacol Ther* 2014, 141:358-367.

13. Wadström BN, Pedersen KM, Wulff AB, Nordestgaard BG: Elevated remnant cholesterol and atherosclerotic cardiovascular disease in diabetes: a population-based prospective cohort study. *Diabetologia* 2023, 66:2238-2249.

14. Wu W, Chen G, Wu K, Zheng H, Chen Y, Wang X, Huang Z, Cai Z, Cai Z, Chen Z, et al: Cumulative exposure to high remnant-cholesterol concentrations increases

the risk of cardiovascular disease in patients with hypertension: a prospective cohort study. *Cardiovasc Diabetol* 2023, 22:258.

15. Tian Y, Wu Y, Qi M, Song L, Chen B, Wang C, Lu J, Yang Y, Zhang X, Cui J, et al: **Associations of remnant cholesterol with cardiovascular and cancer mortality in a nationwide cohort.** *Sci Bull (Beijing)* 2024, **69:**526-534.

16. Wadström BN, Pedersen KM, Wulff AB, Nordestgaard BG: Elevated remnant cholesterol, plasma triglycerides, and cardiovascular and non-cardiovascular mortality. *Eur Heart J* 2023, 44:1432-1445.

17. Chen J, Su Y, Su X, Luo F: Remnant cholesterol has a non-linear association with non-alcoholic fatty liver disease. *Diabetes Res Clin Pract* 2023, 201:110733.

18. Huang H, Guo Y, Liu Z, Zeng Y, Chen Y, Xu C: Remnant Cholesterol Predicts Long-term Mortality of Patients With Metabolic Dysfunction-associated Fatty Liver Disease. *J Clin Endocrinol Metab* 2022, 107:e3295-e3303.

19. Li M, Zhang W, Zhang M, Li L, Wang D, Yan G, Qiao Y, Tang C: Nonlinear relationship between untraditional lipid parameters and the risk of prediabetes: a large retrospective study based on Chinese adults. *Cardiovasc Diabetol* 2024, 23:12.

20. Hou X, Tian F, Guo L, Yu Y, Hu Y, Chen S, Wang M, Yang Z, Wang J, Fan X, et al: Remnant cholesterol is associated with hip BMD and low bone mass in young and middle-aged men: a cross-sectional study. *J Endocrinol Invest* 2024.

21. von Elm E, Altman DG, Egger M, Pocock SJ, Gøtzsche PC, Vandenbroucke JP: The Strengthening the Reporting of Observational Studies in Epidemiology (STROBE) statement: guidelines for reporting observational studies. *Lancet* 2007,

370:1453-1457.

22. **MEC Laboratory Procedures Manual** [https://wwwn.cdc.gov/nchs/data/nhanes/2013-2014/manuals/2013_mec_laboratory_procedures_manual.pdf]

23. Friedewald WT, Levy RI, Fredrickson DS: Estimation of the concentration of low-density lipoprotein cholesterol in plasma, without use of the preparative ultracentrifuge. *Clin Chem* 1972, 18:499-502.

24. Mach F, Baigent C, Catapano AL, Koskinas KC, Casula M, Badimon L, Chapman MJ, De Backer GG, Delgado V, Ference BA, et al: **2019 ESC/EAS Guidelines for the management of dyslipidaemias: lipid modification to reduce cardiovascular risk.** *Eur Heart J* 2020, **41:**111-188.

25. Shepherd JA, Fan B, Lu Y, Wu XP, Wacker WK, Ergun DL, Levine MA: A multinational study to develop universal standardization of whole-body bone density and composition using GE Healthcare Lunar and Hologic DXA systems. *J Bone Miner Res* 2012, 27:2208-2216.

26. **Body Composition Procedures Manual** [https://wwwn.cdc.gov/nchs/data/nhanes/2017-2018/manuals/Body_Composition_Procedures_Manual_2018.pdf]

27. Li Z, Lai J, Wen L, Chen Q, Tan R, Zhong X, Liu Y, Liu Y: Higher Levels of Blood Selenium are Associated with Higher Levels of Serum Lipid Profile in US Adults with CKD: Results from NHANES 2013-2018. *Biol Trace Elem Res* 2023, 201:5501-5511.

28. Xie YY, Zhao L, Gao LJ, Xu RX, Gao Y, Dou KF, Guo YL, He YM: Association between remnant cholesterol and verbal learning and memory function in the elderly in the US. *Lipids Health Dis* 2022, 21:120.

29. Tong J, Li X, Liang X, Tang F, Ren Y, Hao G, Peng X, Luo S, Feng Y, Huang D, et al: **The relationship of remnant cholesterol and abdominal obesity in children: A cross-sectional study.** *Front Cardiovasc Med* 2022, **9:**929560.

30. Miao Y, Tao H: Association between remnant lipoprotein cholesterol levels and risk of non-alcoholic fatty liver disease in non-obese populations: a Chinese longitudinal prospective cohort study. *BMJ Open* 2023, 13:e069440.

31. Skudder-Hill L, Sequeira-Bisson IR, Ko J, Cho J, Poppitt SD, Petrov MS: Remnant cholesterol, but not low-density lipoprotein cholesterol, is associated with intra-pancreatic fat deposition. *Diabetes Obes Metab* 2023, 25:3337-3346.

32. Guan B, Wang A, Xu H: Causal associations of remnant cholesterol with cardiometabolic diseases and risk factors: a mendelian randomization analysis. *Cardiovasc Diabetol* 2023, 22:207.

33. Deng T, Lyon CJ, Bergin S, Caligiuri MA, Hsueh WA: **Obesity, Inflammation, and Cancer.** *Annu Rev Pathol* 2016, **11:**421-449.

34. Lumeng CN, Bodzin JL, Saltiel AR: Obesity induces a phenotypic switch in adipose tissue macrophage polarization. *J Clin Invest* 2007, 117:175-184.

35. Collado A, Marques P, Domingo E, Perello E, González-Navarro H, Martinez-Hervás S, Real JT, Piqueras L, Ascaso JF, Sanz MJ: **Novel Immune Features of the Systemic Inflammation Associated with Primary**

Hypercholesterolemia: Changes in Cytokine/Chemokine Profile, Increased Platelet and Leukocyte Activation. *J Clin Med* 2018, **8**.

36. Maeda N, Funahashi T, Matsuzawa Y, Shimomura I: **Adiponectin, a unique adipocyte-derived factor beyond hormones.** *Atherosclerosis* 2020, **292:**1-9.

20240709220105100817310766638284

ORIGINALITY REPORT

23%
SIMILARITY INDEX

PRIMARY SOURCES

1	www.frontiersin.org<br>Internet	87 words — 2%
2	Xi Gu, Fanfan Zhu, Ping Gao, Ying Shen, Leiqun Lu. "Association between visceral adipose tissue and total testosterone among the United States male adults: a cross-sectional study", International Journal of Impotence Research, 2024<br>Crossref	76 words — 2%
3	lipidworld.biomedcentral.com<br>Internet	59 words — 2%
4	www.mdpi.com<br>Internet	45 words — 1%
5	hdl.handle.net<br>Internet	41 words — 1%
6	www.researchsquare.com<br>Internet	37 words — 1%
7	Wenxue Sun, Junting Liu, Eurídice Martinez Steele, Xin Yang, Ran Gao, Chunping Wang, Junxiu Liu. "Association of ultra-processed food consumption with muscle mass among young and middle-aged US adults", European Journal of Nutrition, 2024<br>Crossref	21 words — 1%

8	coek.info<br>Internet	21 words — 1%
9	bmjopen.bmj.com<br>Internet	20 words — 1%
10	www.portailvasculaire.fr<br>Internet	20 words — 1%
11	Andrej M. Grjibovski, Per Magnus, Anna Midelfart, Jennifer R. Harris. "Epidemiology and Heritability of Astigmatism in Norwegian Twins: An Analysis of Self-Reported Data", Ophthalmic Epidemiology, 2009<br>Crossref	17 words — < 1%
12	academic.oup.com<br>Internet	17 words — < 1%
13	assets-eu.researchsquare.com<br>Internet	17 words — < 1%
14	www.science.gov<br>Internet	17 words — < 1%
15	assets.researchsquare.com<br>Internet	16 words — < 1%
16	uu.diva-portal.org<br>Internet	16 words — < 1%
17	Xiaoqiang Liu, Yisen Huang, Yingxuan Huang, Chanchan Lin, Boming Xu, Yilin Zeng, Peizhong Chen, Xiaobo Liu, Yubin Wang. "Association of Trouble Sleeping with Increased Risk of Gallstone Disease in U.S. Adults: A Cross-Sectional Study of NHANES 2017-2020", Research Square Platform LLC, 2024<br>Crossref Posted Content	15 words — < 1%

18 Olukayode G. Odufuwa, Richard J. Sheppard, Safina Ngonyani, Ahmadi Bakari Mpelepele et al. "House modifications using insecticide treated screening of eave and window as a vector control tool: evidence from a semi-field system in Tanzania and simulated epidemiological impact", Research Square Platform LLC, 2024 — 14 words — < 1%
Crossref Posted Content

19 Sifan Qian, Shoujiang You, Yaming Sun, Qiuyi Wu et al. "Remnant Cholesterol and Common Carotid Artery Intima-Media Thickness in Patients With Ischemic Stroke", Circulation: Cardiovascular Imaging, 2021 — 14 words — < 1%
Crossref

20 Stefano Ciardullo, Alice Oltolini, Rosa Cannistraci, Emanuele Muraca, Gianluca Perseghin. "Sex-related association of NAFLD and liver fibrosis with body fat distribution in the general US population", The American Journal of Clinical Nutrition, 2022 — 14 words — < 1%
Crossref

21 oamjms.eu — 14 words — < 1%
Internet

22 www.researchgate.net — 14 words — < 1%
Internet

23 www.dovepress.com — 12 words — < 1%
Internet

24 www.springermedizin.de — 12 words — < 1%
Internet

25 Jingfei Chen, Yingjie Su, Xin Su, Fei Luo. "Remnant cholesterol has a non-linear association with non- — 11 words — < 1%

alcoholic fatty liver disease", Diabetes Research and Clinical Practice, 2023
Crossref

---

26 www.pharmacompass.com — 10 words — < 1%
Internet

---

27 Aisling B. Heeran, Jessica McCready, Margaret R. Dunne, Noel E. Donlon et al. "Opposing Immune-Metabolic Signature in Visceral Versus Subcutaneous Adipose Tissue in Patients with Adenocarcinoma of the Oesophagus and the Oesophagogastric Junction", Metabolites, 2021 — 9 words — < 1%
Crossref

---

28 Xi Gu, Xun Wang, Sujie Wang, Ying Shen, Leiqun Lu. "Composite Dietary Antioxidant Index is inversely associated with visceral adipose tissue area among U.S. adults: a cross-sectional study.", Nutrition Research, 2024 — 9 words — < 1%
Crossref

---

29 pubmed.ncbi.nlm.nih.gov — 9 words — < 1%
Internet

---

30 static.frontiersin.org — 9 words — < 1%
Internet

---

31 Da-Chuan Guo, Jing-Wei Gao, Xiang Wang, Zhi-Teng Chen et al. "Remnant cholesterol and risk of incident hypertension: a population-based prospective cohort study", Hypertension Research, 2024 — 8 words — < 1%
Crossref

---

32 Kathleen E. Bainbridge, Danita Byrd-Clark. "Prescription Medication Use and Phantom Odor Perception Among US Adults", Chemosensory Perception, 2019 — 8 words — < 1%
Crossref

33	Xi Gu, Xun Wang, Sujie Wang, Ying Shen, Leiqun Lu. "Composite Dietary Antioxidant Index is inversely associated with visceral adipose tissue area among U.S. adults: A cross-sectional study", Nutrition Research, 2024<br>Crossref	8 words — < 1%
34	discovery.dundee.ac.uk<br>Internet	8 words — < 1%
35	eprint.ncl.ac.uk<br>Internet	8 words — < 1%
36	research-information.bris.ac.uk<br>Internet	8 words — < 1%
37	stacks.cdc.gov<br>Internet	8 words — < 1%
38	topsecretapiaccess.dovepress.com<br>Internet	8 words — < 1%
39	www.drperlmutter.com<br>Internet	8 words — < 1%
40	www.imrpress.com<br>Internet	8 words — < 1%
41	www.peeref.com<br>Internet	8 words — < 1%
42	Connie W. Tsao, Aaron W. Aday, Zaid I. Almarzooq, Cheryl A.M. Anderson et al. "Heart Disease and Stroke Statistics—2023 Update: A Report From the American Heart Association", Circulation, 2023<br>Crossref	7 words — < 1%

43	Lifang Li, Vanessa Hou Cheng Chou, Oscar Hou In Chou, Sakshi Roy et al. "The association between time-weighted remnant cholesterol and cardiovascular and non-cardiovascular mortality: A population-based cohort study", Cold Spring Harbor Laboratory, 2024<br>Crossref Posted Content	7 words — < 1%
44	Xiaoran Bian, Yonghao Zhang, Min Shao, Jiachen Li, Jiaju Ge, Zhuofan Li, Hao Peng, Mingzhi Zhang. "Remnant cholesterol and risk of major adverse cardiovascular events: a systematic review and dose–response meta-analysis of cohort studies", Coronary Artery Disease, 2024<br>Crossref	7 words — < 1%
45	Samira Saghafi, Elham Chamani, Fatemeh Salmani, Reza Fadaei, Efat Shafiei, Nariman Moradi, Tahmine Tavakoli. "Genetic predisposition to nonalcoholic fatty liver disease: insights from ANGPTL8 gene variants in Iranian adults", Lipids in Health and Disease, 2023<br>Crossref	6 words — < 1%
46	Xiaowan Li, Lanyu Wang, Min Liu, Hongyi Zhou, Hongyang Xu. "Association between neutrophil-to-lymphocyte ratio and diabetic kidney disease in type 2 diabetes mellitus patients: a cross-sectional study", Frontiers in Endocrinology, 2024<br>Crossref	6 words — < 1%
47	ir.ymlib.yonsei.ac.kr<br>Internet	6 words — < 1%

EXCLUDE QUOTES	OFF	EXCLUDE SOURCES	OFF
EXCLUDE BIBLIOGRAPHY	ON	EXCLUDE MATCHES	OFF
